# Probing Interfacial Thermal Conductance Under Extremely Large Temperature Differences via Ultrafast X-Ray Diffraction

**DOI:** 10.3390/nano16211360

**Published:** 2026-10-25

**Authors:** Shijie Duan, Jiahao Chen, Changqin Liu, Xiaojuan Yuan, Yifei Li, Jinguang Wang, Xin Lu, Dongdong Kang, Jiayu Dai, Xiaoxiang Yu

**Affiliations:** 1 College of Science, National University of Defense Technology, Changsha 410073, China; 2 Hunan Key Laboratory of Extreme Matter and Applications, National University of Defense Technology, Changsha 410073, China; 3 Hunan Research Center of the Basic Discipline for Physical States, National University of Defense Technology, Changsha 410073, China; 4 Beijing National Laboratory of Condensed Matter Physics, Institute of Physics, Chinese Academy of Sciences, Beijing 100190, China; 5 School of Physical Sciences, University of Chinese Academy of Sciences, Beijing 101408, China

**Keywords:** interfacial thermal conductance, ultrafast X-ray diffraction, extremely large temperature difference

## Abstract

Probing the interfacial thermal transport under extremely large temperature difference (ELTD) is critical for thermal protection and heat dissipation in extreme environments yet challenging. However, conventional optical thermoreflectance methods work only over narrow temperature ranges and thus fail for ELTD conditions characterized by a broad temperature evolution range. Here, we circumvent this limitation by employing a table-top ultrafast X-ray diffraction (UXRD) system, which exploits the nearly linear lattice thermal expansion over a wide temperature range. We investigate interfacial thermal transport in a W thin film on a MgO substrate under ELTD conditions and determine that the interfacial thermal conductance (ITC) value increases from 6.43 × 10^8^ W m^−2^ K^−1^ to 8.71 × 10^8^ W m^−2^ K^−1^ as the maximum temperature of W rises from 670 K to 2334 K, accompanied by an increase in the maximum interface temperature difference from 302 K to 1451 K based on the finite volume method (FVM). The results reside at a relatively high level among reported ITC values for conventional metal-oxide interfaces, which may originate from the simultaneous increase in interface temperature difference and mean interface temperature. This work demonstrates UXRD as an effective tool for ELTD thermal measurements and fills a critical knowledge gap regarding interfacial thermal transport under extreme conditions.

## 1. Introduction

Extremely large temperature difference (ELTD) conditions arise in particular engineering scenarios including spacecraft [1,2,3,4], laser processing [5,6,7,8,9,10], and high-power electronics [11,12,13,14]. Thermal transport under ELTD is critical to thermal protection, manufacturing quality, and heat dissipation. The extensive presence of interfaces between dissimilar materials in devices and equipment introduces additional scattering of heat carriers, giving rise to an extra thermal resistance that makes interfacial thermal conductance (ITC) a key parameter governing energy transfer [15,16,17,18,19,20,21]. The ELTD condition spans a broad temperature evolution range, posing substantial challenges to experimental probing. Notably, a large temperature drop across the interface is physically distinct from a high interface temperature. The ELTD condition would lead to simultaneous increase in interface temperature difference and mean interface temperature. While the effects of interface temperature on ITC have been extensively studied [22,23,24,25,26]—e.g., higher temperatures generally enhance ITC by enhancing interfacial phonon scattering and transmission—the influence of the temperature difference across the interface remains experimentally unexplored. Thus, there is an urgent need to measure ITC under ELTD conditions.

For a typical interfacial thermal transport issue of a metal thin film on an insulator substrate, ELTD conditions are generally realized by ultrafast laser heating. The two-temperature model (TTM) [17,27] is usually adopted to describe the energy transfer in a metal/non-metal interface system after laser heating. Nonequilibrium states emerge on the ultrafast timescale, featuring laser-heated electrons alongside cold lattice phonons [9,10,28]. Temporally, the thermal transport process across the interface occurs after electron and phonon subsystems in metal reach equilibrium [29,30]. Such a temporal window is of primary interest for experimental thermal characterization.

The primary technical challenge lies in accurately measuring temperature over a broad temperature range as the interface system evolves from ELTD back to ambient conditions. Pump–probe thermoreflectance methods, represented by time-domain and frequency-domain thermoreflectance, have long been the mainstream approaches for thermal characterization [31,32,33,34,35]. In these techniques, temperature is deduced from optical reflectivity changes under the assumption of a constant thermoreflectance coefficient within a small temperature rise [31,32,33,34]. However, under ELTD, the large rise and wide range of temperature invalidate the linear reflectivity–temperature relationship for transducer metals [33]. For instance, the thermoreflectance coefficient of aluminum has varied by almost an order of magnitude from room temperature to 600 K [36] and its low melting point (~933 K [37]) prevents its use at high temperatures [38]. Platinum can endure higher temperatures, yet its thermoreflectance coefficient is small [36]. Titanium is prone to thermomechanical failure under ELTD conditions [39]. Low-cost transient thermal measurement methods [40] feature small temperature rises and bulk-sample geometry. Consequently, experimental approaches capable of probing ITC at ELTD are still lacking.

In this work, we overcome this limitation by establishing a table-top ultrafast X-ray diffraction (UXRD) system. Unlike optical thermoreflectance, UXRD can serve as a probing source in pump–probe thermal measurements by tracking diffraction pattern changes induced by lattice thermal expansion [41,42,43,44,45,46,47,48,49]. This technique has been employed to quantify phonon transport across interfaces and to monitor nanoscale heat diffusion with exceptional sensitivities [50,51]. More recently, ultrafast electron diffraction (UED) combined with machine learning has extended these capabilities to frequency-resolved mapping of interfacial phonon dynamics [18]. However, such UXRD and UED approaches have not been applied to the extremely wide temperature range required under ELTD conditions. Importantly, the variation in the coefficient of linear thermal expansion ($\alpha_{l}$) over a wide temperature range is usually much smaller than that of the coefficient of thermoreflectance [52,53]. For example, $\alpha_{l}$ of tungsten (W) changes from ~4.5 × 10^−6^ K^−1^ to ~6.2 × 10^−6^ K^−1^ as the temperature rises from room temperature to ~2000 K [53], ensuring reliable lattice temperature extraction. Using UXRD, we investigate interfacial thermal transport in a W thin film grown on a MgO substrate under ELTD conditions. Based on the lattice temperature evolution measured by UXRD, we construct a one-dimensional transient heat conduction model using the finite volume method (FVM), delivering the full spatiotemporal temperature distributions along the film depth as a function of delay time. By iteratively fitting the simulated temperature evolution curves of the W film to the experimental measurements, we quantitatively extract the ITC values. The obtained ITC increases from 6.43 × 10^8^ W m^−2^ K^−1^ to 8.71 × 10^8^ W m^−2^ K^−1^ as the maximum temperature of W rises from 670 K to 2334 K, accompanied by an increase in the maximum interface temperature difference from 302 K to 1451 K based on the finite volume method (FVM). This work demonstrates that UXRD is an effective tool for ELTD thermal measurements and fills a critical knowledge gap regarding interfacial phonon transport under ELTD conditions.

## 2. Materials and Methods

### 2.1. Sample Preparation and Characterization

**Figure A1:**
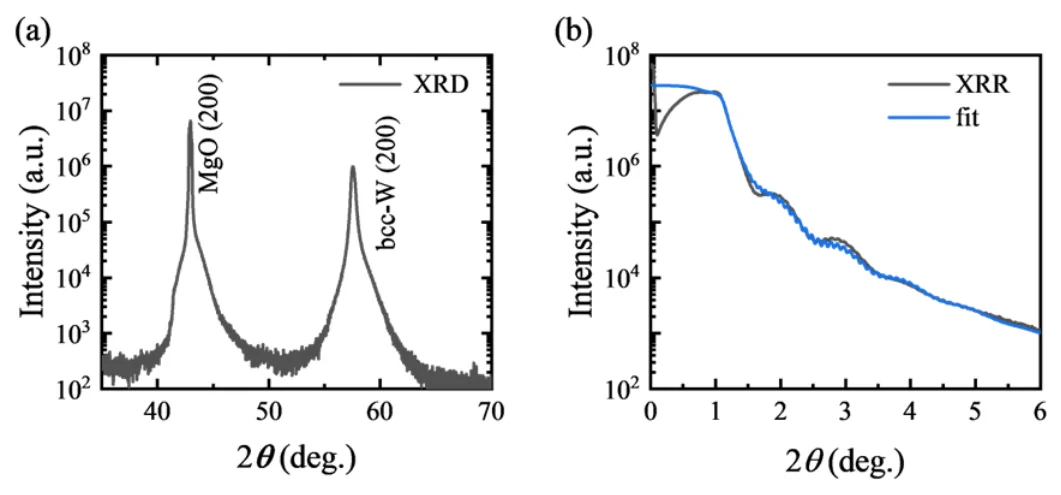
(**a**) XRD and (**b**) XRR characterization of W/MgO.

The W (200) epitaxial films were grown on 500 μm thick MgO (200) substrates by DC magnetron sputtering at a base pressure of 8.6 × 10^−6^ Pa. The substrate was pre-annealed at 700 °C for 1 h. The W film was then deposited at 700 °C under a sputtering pressure of 0.4 Pa using a 99.999% pure W target. After deposition, the samples were cooled to room temperature in vacuum, followed by the in situ deposition of a 10 nm MgO capping layer. The Rigaku SmartLab diffractometer was used to measure the X-ray diffraction (XRD) and X-ray reflectivity (XRR). As shown in Figure A1a, the XRD signals identified that the Bragg peaks of W (200) and MgO (200) were at 2*θ_b_W_*_(200)_ = 57.54° and 2*θ_b_MgO_*_(200)_ = 42.97°, respectively. The measured lattice constant of W is 3.20 Å, 1.1% larger than the bulk value (3.165 Å [54]), indicating the presence of out-of-plane tensile residual strain in the thin film. The XRR patterns of the samples are presented in Figure A1b. By utilizing the SmartLabStudioII software to fit the data, we can determine that the thickness of W film is 82.0 ± 0.5 nm (see Table A1).

### 2.2. Ultrafast X-Ray Diffraction

**Figure 1:**
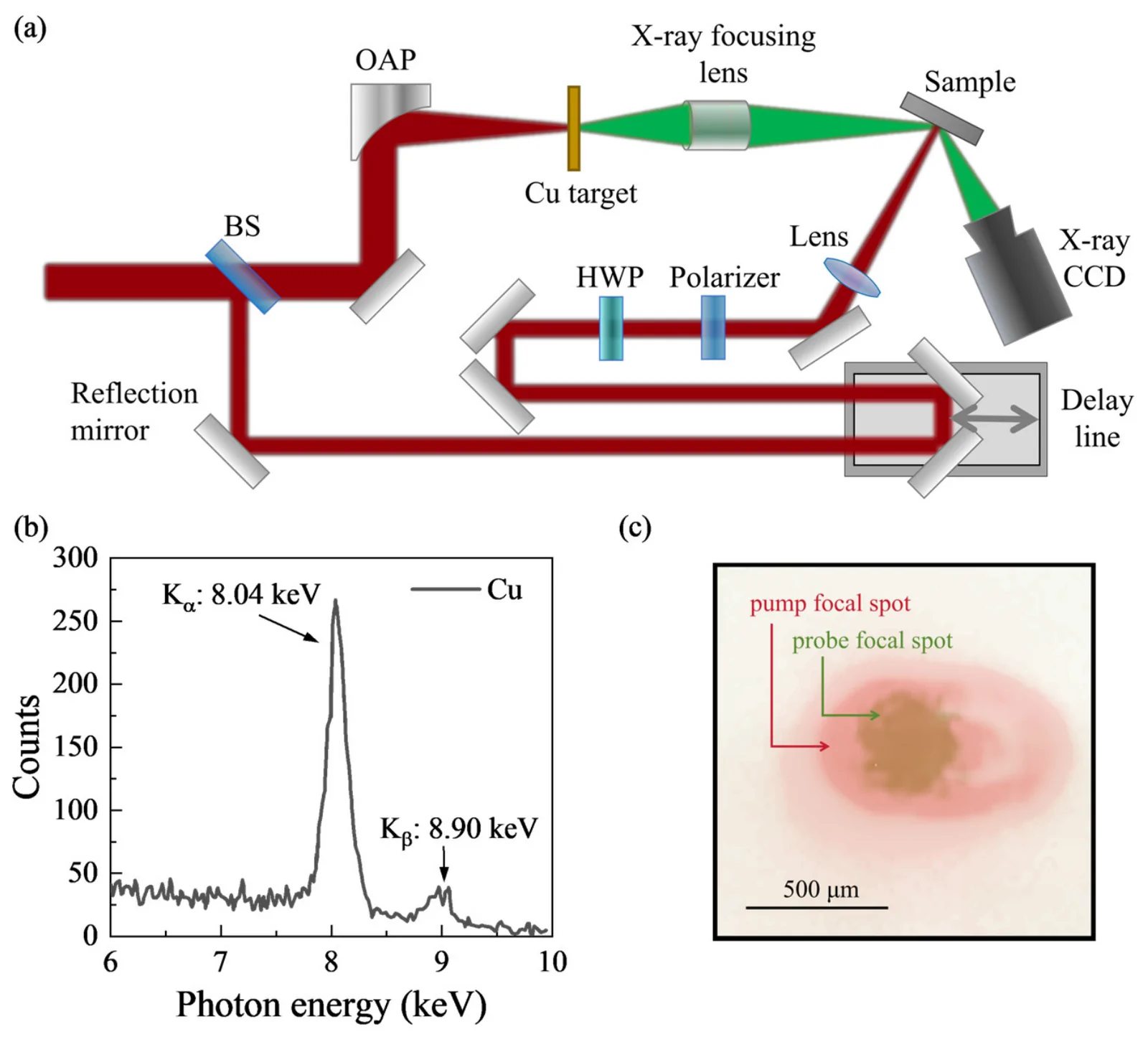
(**a**) Schematic diagram of UXRD system. BS: beam splitter. HWP: half-wave plate. OAP: off-axis parabolic mirror. (**b**) The spectrum of laser-induced X-ray radiation generated on the Cu target. (**c**) The relative position and size comparison between the pump spot and the X-ray probe footprint.

The UXRD system was driven by a femtosecond laser system with peak power of 3 TW, pulse duration below 30 fs, central wavelength of 800 nm and repetition rate of 100 Hz. As shown in Figure 1, the laser pulse was split into two beams by a beam splitter (BS). The weaker pulse, serving as the pump laser, was directed onto a mechanical delay stage and finally focused on the sample with an elliptical spot size of 922 μm × 512 μm (1/e^2^ diameter). The pump fluence was tunable by using a half-wave plate (HWP) and a polarizer. The main pulse was focused on the surface of the copper (Cu) tape target by a small-focal-length off-axis parabolic (OAP) mirror to generate K_α_ X-ray radiation, which served as the probe beam. The probe focal spot exhibits a nearly circular Gaussian profile with 1/e^2^ diameters of 376 μm × 376 μm. The probe footprint is smaller than the pump spot, ensuring pump excitation across the probed region. The X-ray CCD (1340 × 400 pixels of 20 × 20 μm^2^ size) was positioned at the W (200) Bragg peak with an exposure time of 30 s at each time delay. By controlling the time delay between the pump and probe pulse, we acquire a series of time-resolved diffraction patterns. By performing Gaussian-fits to the integrated intensity along the *x*-axis, we obtain time-dependent diffraction peak shifts (Δθ).

### 2.3. The Two-Process Model for Peak-Shift Fitting

The shift in the Bragg diffraction peak originates from both thermal and acoustic wave contributions.

The thermal contribution of Δθ is fitted by

(1) $$f_{th}(t)=-A_{th}\left(1-e^{-(t-t_{0})/\tau_{ep}}\right)e^{-(t-t_{0})/\tau_{th}}H(t-t_{0})$$

where $A_{th}$ is the amplitude, $t_{0}$ is the time zero, $\tau_{ep}$ is the electron–phonon coupling (EPC) relaxation time describing the initiation of lattice expansion resulting from the lattice temperature rise, $\tau_{th}$ is the thermal diffusion time describing the recovery of lattice expansion owing to the lattice temperature recovery, and H(t) is the Heaviside step function.

The acoustic wave contribution is fitted by

(2) $$f_{aw}(t)=-A_{aw}e^{-\frac{t-t_{0}}{\tau_{aw}}}\sin\left(\frac{2\pi \left(t-t_{0}\right)}{P}\right)H(t-t_{0})$$

where $A_{aw}$ is the amplitude, $\tau_{aw}$ is the amplitude decay time, and P is the oscillation period.

### 2.4. Thermal Diffusion Model

The W film thickness (D=82 nm) in this experiment is comparable to the electron mean free path of W (40–54 nm [55,56,57]). The phonon thermal conductivity of W exhibits a pronounced size effect below 100 nm: when the film thickness becomes comparable to the mean free path of heat-carrying phonons, the thermal conductivity decreases gradually with decreasing thickness, suggesting a phonon mean free path of ~100 nm for W [58]. The heat carrier transport thus falls within the ballistic regime and the temperature gradient inside the W film can be neglected [59,60].

To accurately reproduce the actual heat transport process and capture the temperature evolution inside the MgO substrate as well as the temperature jump at the interface, a one-dimensional transient heat conduction numerical model is established in this work, with spatial discretization by the FVM and time integration by the explicit Euler scheme. The energy of the femtosecond laser is converted into lattice thermal energy of W through EPC, which is equivalent to a uniformly distributed exponentially decaying volume heat source in W. The governing equation inside the W layer is

(3) $$C_{W}\frac{{dT}_{W}\left(t\right)}{dt}=p\left(t\right)-\frac{G}{D}\left(T_{W}(t)-T_{MgO}(D,t)\right),$$

where $C_{W}$ is the volumetric heat capacity of W and $p\left(t\right)$ is the volume heat source power density, satisfying $p\left(t\right)=p_{0}\exp\left(-t/\tau_{\mathrm{ep}}\right)$, where the peak power density $p_{0}$ is calibrated by the total laser absorption energy in the experiment. We employ the average heat capacity over the experimentally extracted cooling profile from maximum temperature to 200 ps, as the heat capacity varies weakly within this range. The governing equation inside the MgO layer is

(4) $$C_{\mathrm{MgO}}\frac{\partial T_{MgO}\left(z,t\right)}{\partial t}=\kappa_{\mathrm{MgO}}\frac{\partial^{2}T_{MgO}\left(z,t\right)}{\partial z^{2}},$$

where $C_{\mathrm{MgO}}$ and $\kappa_{\mathrm{MgO}}$ are the volumetric heat capacity and thermal conductivity of MgO, respectively. At the interface (z=D), the heat flux is continuous and satisfies the Kapitza interfacial heat transport relation:

(5) $$\kappa_{\mathrm{MgO}}{\left.\frac{\partial T_{MgO}}{\partial z}\right|}_{z=D}=-G\left(T_{W}(t)-T_{MgO}(D,t)\right).$$

An adiabatic boundary condition is imposed at the top surface of the W film (z=0). At the bottom of the MgO substrate ($z=D+d_{\mathrm{MgO}}$), a constant temperature boundary condition is adopted and maintained at the initial room temperature: $T\left(D+d_{\mathrm{MgO}},t\right)=T_{0}$. Initial conditions include $T_{W}\left(0\right)=T_{0}$ and $T_{MgO}\left(z,0\right)=T_{0}$.

Spatial discretization is performed using the FVM, with nodes located at the center of each control volume. The W layer and MgO layer are meshed independently. Two adjacent nodes are set at the interface corresponding to the W side and MgO side, respectively. The discretization scheme naturally satisfies energy conservation. Time integration adopts the explicit Euler scheme, and the time step is constrained by both the intra-layer heat diffusion stability condition and the interfacial heat flow stability condition,

(6) $$\Delta t<\min\left(\frac{C_{W}D}{2G},\frac{C_{MgO}\Delta z}{2G},\frac{C_{MgO}\Delta z^{2}}{2\kappa_{MgO}}\right),$$

where Δz is the grid step size of the MgO layer. In actual calculation, 0.4 times the upper stability limit is taken as the time step to ensure absolute numerical stability.

## 3. Results

### 3.1. Temporal Evolution of Diffraction Peak Shift

**Figure 2:**
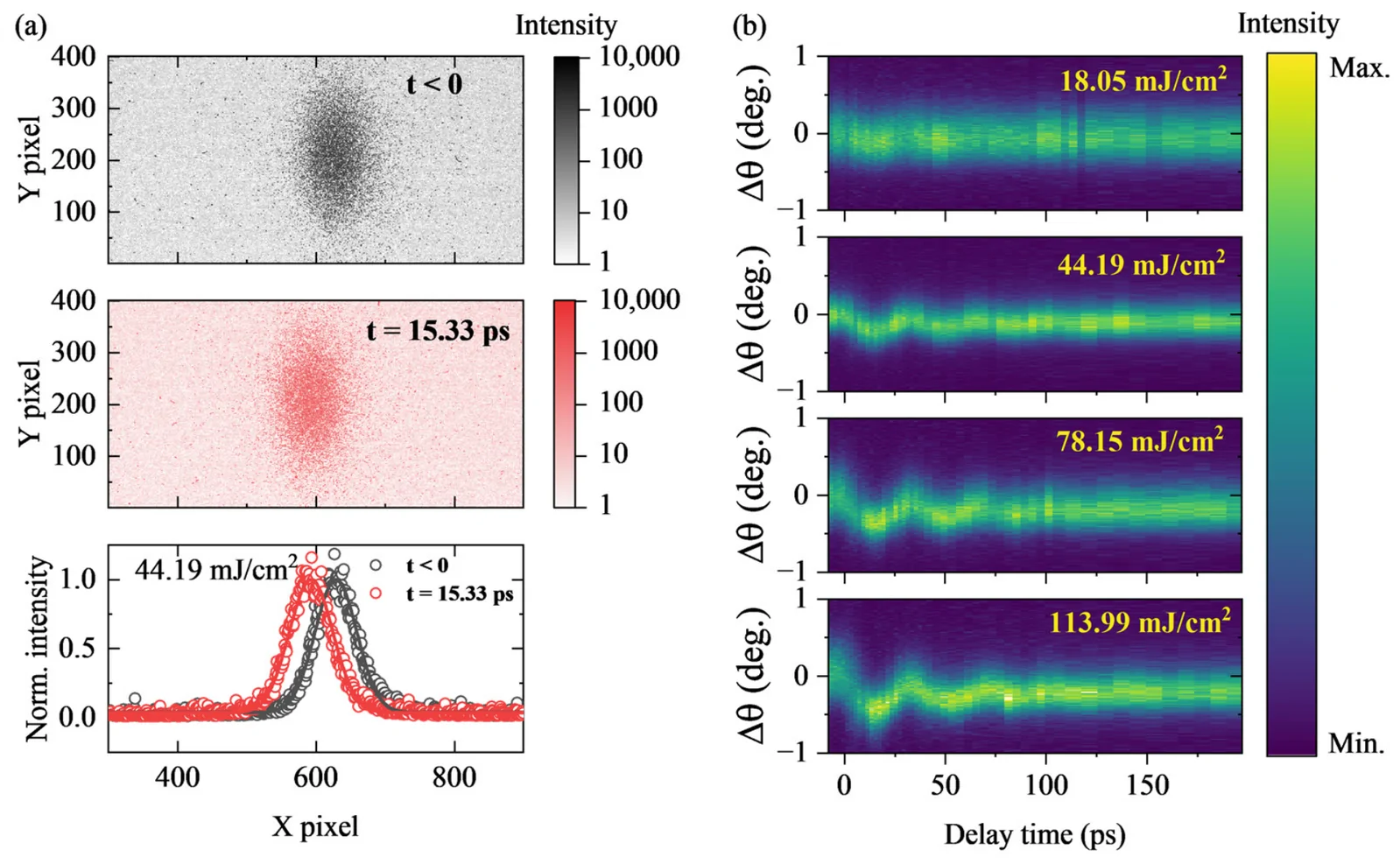
(**a**) Diffraction patterns before and after time zero and their integrated data with Gaussian-fitting results under the pump fluence of 44.19 mJ/cm^2^. (**b**) Integrated data at each time delay under pump fluences of 18.05, 44.19, 78.15 and 113.99 mJ/cm^2^.

**Figure 3:**
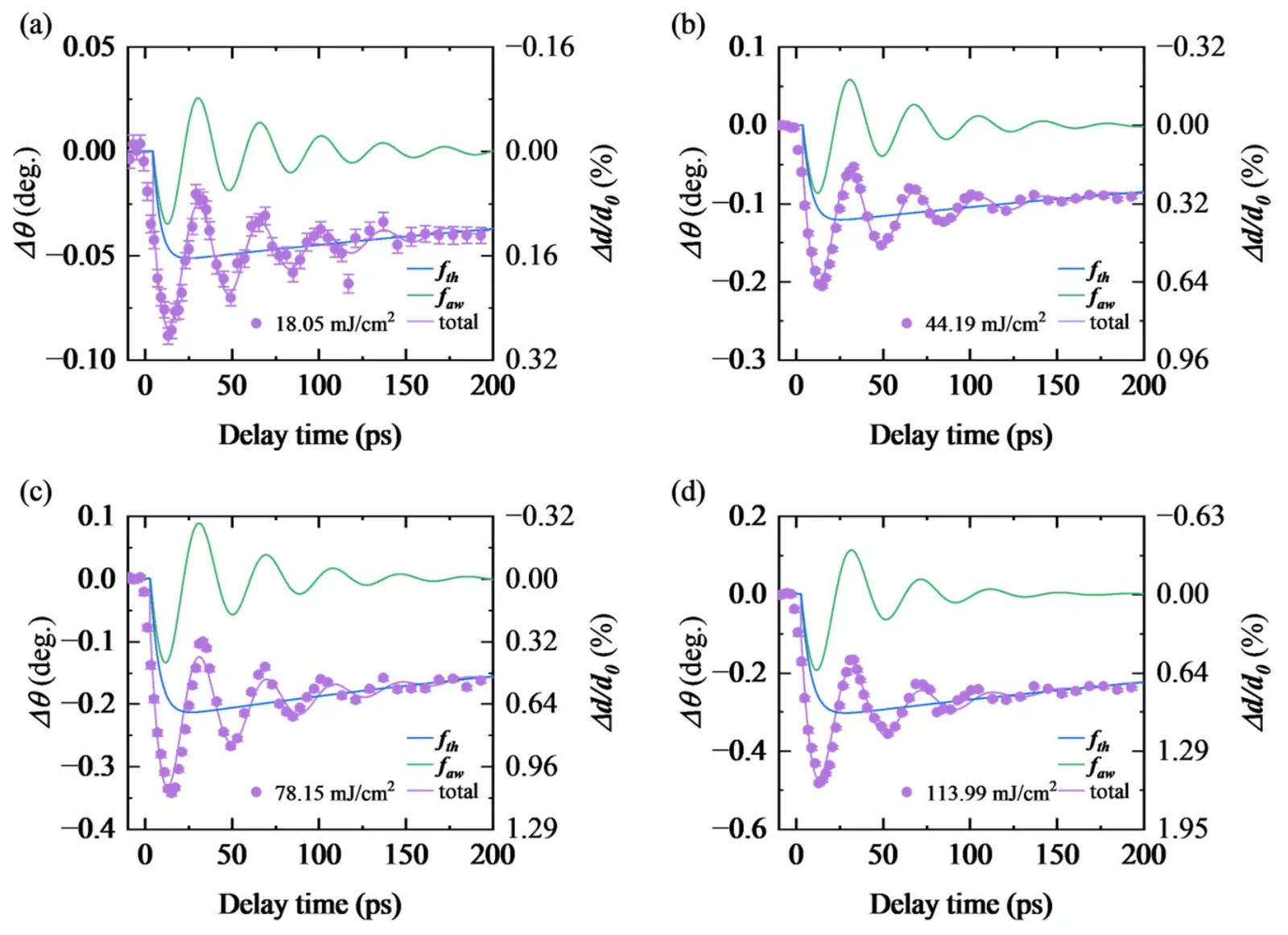
Temporal evolution of diffraction peak shift (Δθ) and fitting results under pump fluences of (**a**) 18.05, (**b**) 44.19, (**c**) 78.15 and (**d**) 113.99 mJ/cm^2^. The thermal contribution and acoustic wave contribution are denoted as $f_{th}$ and $f_{aw}$, respectively.

Figure 2a shows diffraction patterns recorded by CCD before and after time zero and their integrated data with Gaussian-fitting results under the pump fluence of 44.19 mJ/cm^2^. Figure 2b shows integrated results at each time delay under pump fluences from 18.05 to 113.99 mJ/cm^2^. No irreversible change in the intrinsic properties of the sample is observed before and after multiple experimental cycles. The temporal evolution of diffraction peak shift (Δθ) is shown as purple dots in Figure 3. The peak position starts to shift from $\theta_{0}$ toward lower angles after time zero, reaching its maximum variation at ~15 ps and then gradually recovers. The negative change in peak position corresponds to positive lattice parameter change ($\Delta d/d_{0}$).

Δθ arises from two primary contributions: thermal expansion and relaxation induced by lattice temperature variations, as well as the propagation and dissipation of acoustic waves. We adopt a two-process model (see Section 2.3) to fit the experimental data [61,62], with the fitting curves plotted as purple lines in Figure 3. The thermal contribution ($f_{th}$) and acoustic wave contribution ($f_{aw}$) are depicted as blue and green lines, respectively. Under the pump fluence of 113.99 mJ/cm^2^, the maximum $\Delta d/d_{0}$ even reaches 1.60%. Δθ has not returned to zero at 200 ps, indicating that the thermal dissipation process is still ongoing. The oscillation period P satisfies the relation $v_{l}=2D/P$, where $v_{l}$ is the longitudinal acoustic velocity of W.

### 3.2. Estimation of Lattice Temperature of W

**Figure 4:**
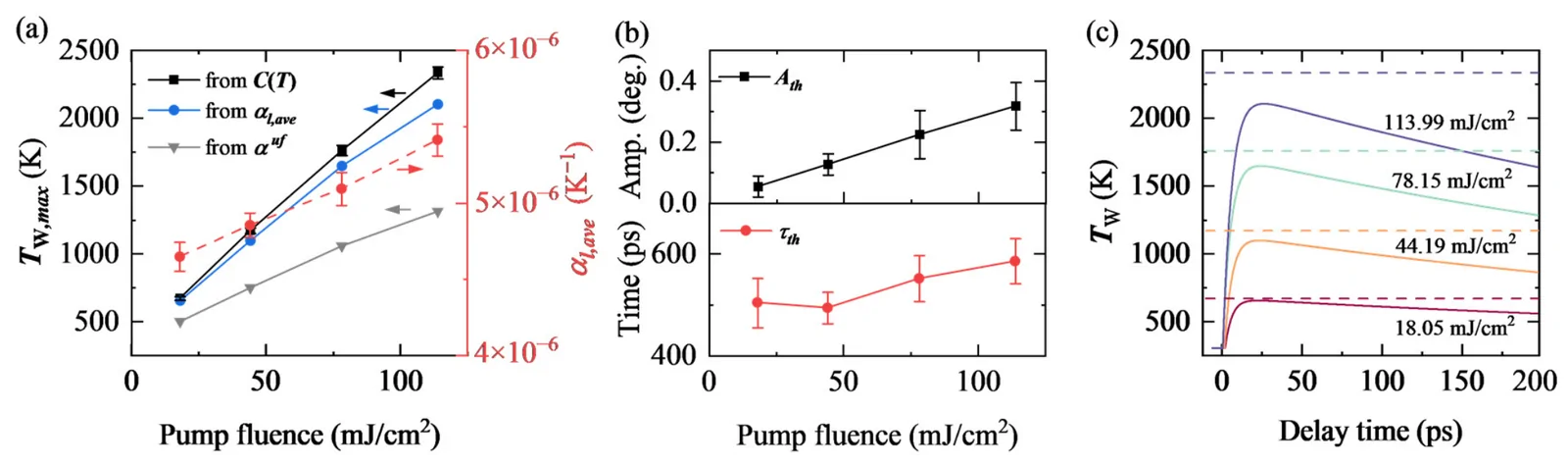
(**a**) Left axis: estimated $T_{W,max}$ from volumetric heat capacity C(T) (black line), average linear thermal expansion coefficient $\alpha_{l,ave}$ (blue line), and ultrafast expansion coefficient $\alpha^{uf}$ (light gray line) under different pump fluences. Right axis: $\alpha_{l,ave}$ under different pump fluences. (**b**) The amplitude ($A_{th}$) and time constant ($\tau_{th}$) of the thermal diffusion component under different pump fluences. (**c**) Temporal evolution of lattice temperature of W derived from the UXRD measurement. Dashed lines indicate $T_{W,max}$ estimated from C(T).

$f_{th}$ reflects the thermal expansion related to the evolution of lattice temperature of W, $T_{W}$. As suggested in TTM, the electron temperature in W rises immediately after laser heating, and then $T_{W}$ rises from the room temperature ($T_{0}$) to its maximum ($T_{W,max}$) via EPC on the picosecond timescale [9,10,63], followed by a slow recovery. First, we estimate $T_{W,max}$ from volumetric heat capacity C(T) [64] and optical properties [37,65] of W by $\Delta Q=\int_{T_{0}}^{T_{W,max}}C(T)dT$, where ΔQ is the total deposited energy per volume in the film. $\Delta Q=(1-R)F\left(1-\exp(-D/\delta )\right)/D$, where R≈0.55 is the reflectivity at 800 nm, F is the pump fluence in our experiment, and δ is the optical penetration depth [65]. As shown in Figure 4a, $T_{W,max}$ estimated from C(T) rises from 670 K to 2334 K with increasing pump fluences.

The $\alpha_{l}$ grows weakly from room temperature to 2334 K [53]. Here we treat $\alpha_{l}(T)$ as a constant. Thus, as marked by the red dots in Figure 4a, we extract the average value of $\alpha_{l}$ within each temperature interval by $\alpha_{l,ave}=\int_{T_{W,1}}^{T_{W,max}}\alpha_{l}\left(T\right)dT/(T_{W,max}-T_{W,1})$, where $T_{W,1}$ is the temperature at the end of the probing time window. $T_{W,max}$ can also be derived from $\alpha_{l,ave}$ by ${\left(\Delta d/d_{0}\right)}_{th,max}=\alpha_{l,ave}\left(T_{W,max}-T_{W,1}\right).$ Figure 4a presents $T_{W,max}$ derived from C(T) and $\alpha_{l,ave}$. For an ideal fully clamped continuous W film, Poisson effect modification yields an ultrafast out-of-plane thermal expansion coefficient $\alpha^{uf}=\frac{1+\nu }{1-\nu }\alpha_{l}\approx 1.78\;\alpha_{l}$ [47,49], where ν is Poisson’s ratio [37]. As shown in Figure 4a, adopting the ideal fully clamped $\alpha^{uf}$ produces temperatures inconsistent with our energy-based calculation. The defects and residual tensile strain may modify the intrinsic $\alpha_{l}$ of W film. The interface roughness may affect the clamping effect of the substrate, and thus may deviate from the ideal fully clamped model. The agreement between maximum temperatures extracted based on $\alpha_{l,ave}$ and heat capacity probably stems from the possible modification of intrinsic $\alpha_{l}$ and the deviation from the ideal model. As shown in Figure 4b, $A_{th}$ grows linearly with increasing pump fluences. $\tau_{th}$ increases from ~500 ps to ~580 ps, which will be used to derive G later. We deduce the temporal evolution of $T_{W}$ from $f_{th}$, as shown in Figure 4c, demonstrating the effectiveness of UXRD in ELTD thermal measurements.

### 3.3. Determination of Interfacial Thermal Conductance

We now focus on the thermal diffusion process in the sample. Since the diameter of the laser spot is hundreds of micrometers (see Section 2.2), much larger than the thickness of W film, the contribution of in-plane heat conduction can be neglected. Thus, heat flow can be considered one-dimensional along the out-of-plane direction [66]. Moreover, the temperature gradient within the film can be neglected (see Section 2.4). Therefore, the temperature gradient exists primarily at the interface between W and MgO. We consider a one-dimensional heat flow along the *z*-axis involving a laser-heated W thin film with thickness D on a semi-infinite MgO substrate. The laser-heated area of the W film has a uniform temperature $T_{W}(t)$. Heat diffuses across the interface into the MgO substrate, forming a temperature distribution $T_{MgO}(z,t)$ in the MgO substrate. We introduce the ITC, G, to describe the heat transfer across the W/MgO interface.

**Figure 5:**
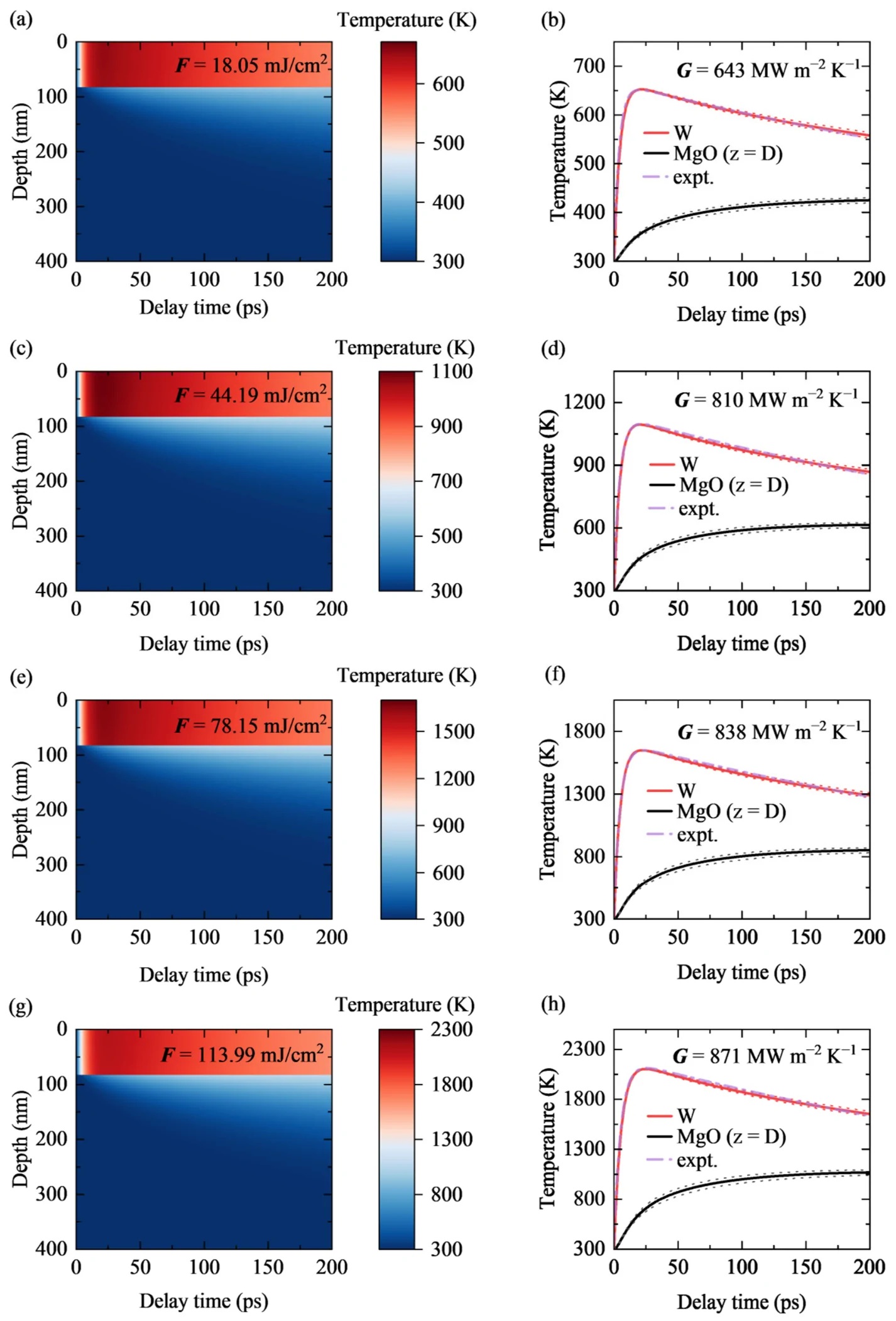
FVM numerical results of spatiotemporal temperature evolution of W/MgO system under different pump fluences. Panels (**a**,**c**,**e**,**g**) show the temperature distribution along the depth direction as a function of delay time at pump fluences of 18.05, 44.19, 78.15, and 113.99 mJ/cm^2^, respectively. Panels (**b**,**d**,**f**,**h**) present the corresponding temperature evolution curves: red solid lines for the W film, black solid lines for the MgO side at the interface, and purple dash–dot lines for experimental measurements. The red and black dotted lines are the fitted temperature profiles when G fluctuates by ±10% relative to the optimal fitting value.

To quantitatively determine G under different pump fluences, we perform one-dimensional transient heat conduction simulations based on the FVM described in Section 2.4. By iteratively tuning the value of G, the simulated temperature relaxation curve of W is fitted to the experimental measurement over the entire time range, yielding the realistic ITC under each pump fluence. Figure 5 presents the temperature distribution as a function of delay time and depth, and displays the temperature evolution curves of W (red solid lines) and at the MgO interface (black solid lines), together with the experimentally extracted data (purple dash–dot lines).

**Figure A2:**
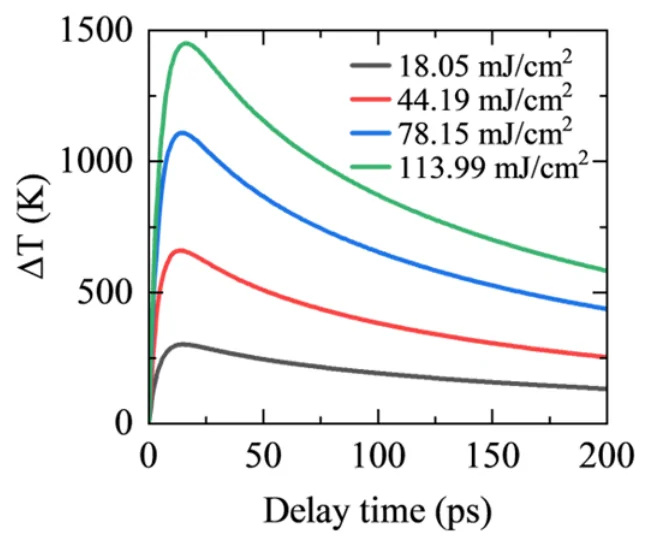
The temporal evolution of the interface temperature difference under different pump fluences.

At the lowest pump fluence of 18.05 mJ/cm^2^, the optimal fitted G is 6.43 × 10^8^ W m^−2^ K^−1^. When the W film reaches a peak temperature around 650 K, the temperature at the MgO side of the interface rises to ~360 K and gradually to ~420 K at 200 ps. At the highest fluence of 113.99 mJ/cm^2^, the optimal fitted ITC G reaches 8.71 × 10^8^ W m^−2^ K^−1^. When the W film reaches a peak temperature around 2100 K, the temperature at the MgO side of the interface rises to ~750 K and gradually to ~1050 K at 200 ps. The region with pronounced temperature rise extends to a depth of roughly 100 nm inside the MgO substrate. For all fluences, the simulated temperature relaxation curves of the W film show good agreement with the experimental measurements, confirming the reliability of the fitted ITC values. The red and black dotted lines in the right panels of Figure 5 represent the fitting results when G fluctuates by ±10% from the optimal value, reflecting the parameter sensitivity of the fitting. The temporal evolution of the interface temperature difference is shown in Figure A2.

**Figure A3:**
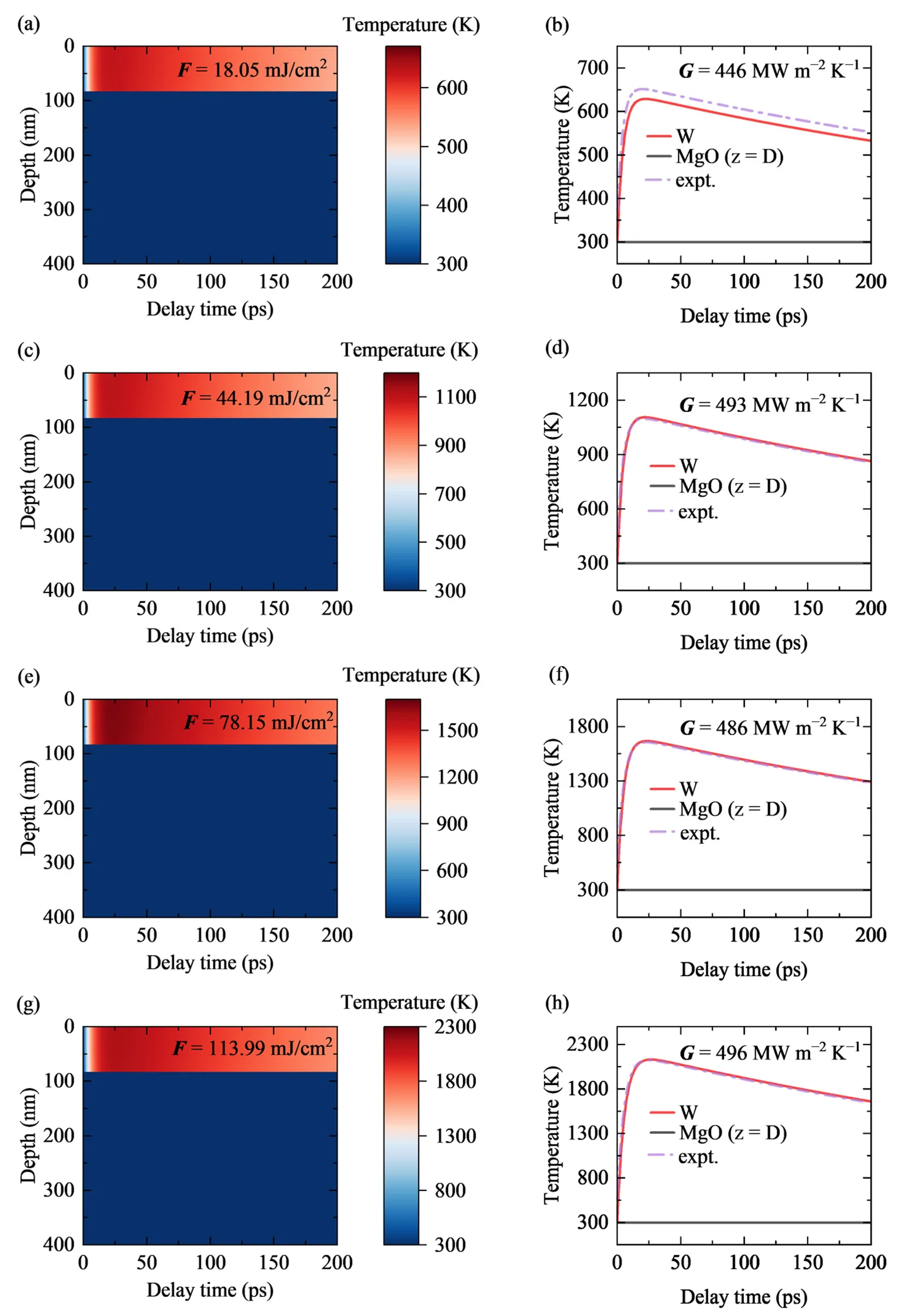
FVM numerical results of spatiotemporal temperature evolution under the ideal heat sink assumption with negligible temperature rise in the MgO substrate. Panels (**a**,**c**,**e**,**g**) show the temperature distribution along the depth direction as a function of delay time at pump fluences of 18.05, 44.19, 78.15, and 113.99 mJ/cm^2^, respectively. Panels (**b**,**d**,**f**,**h**) present the corresponding temperature evolution curves: red solid lines for the W film, black solid lines for the MgO side at the interface, and purple dash–dot lines for experimental measurements.

For comparison, we also evaluate the ITC under the ideal heat sink approximation, in which the MgO substrate is assumed to have infinite heat capacity and thermal conductivity so that its temperature rise can be neglected. Under this assumption, the interface temperature difference driving the interfacial heat flow is entirely determined by the temperature rise in the W film. This approximation inherently leads to an underestimation of ITC due to the overestimation of interface temperature difference. The detailed calculation results under the ideal heat sink approximation are provided in Figure A3.

**Figure 6:**
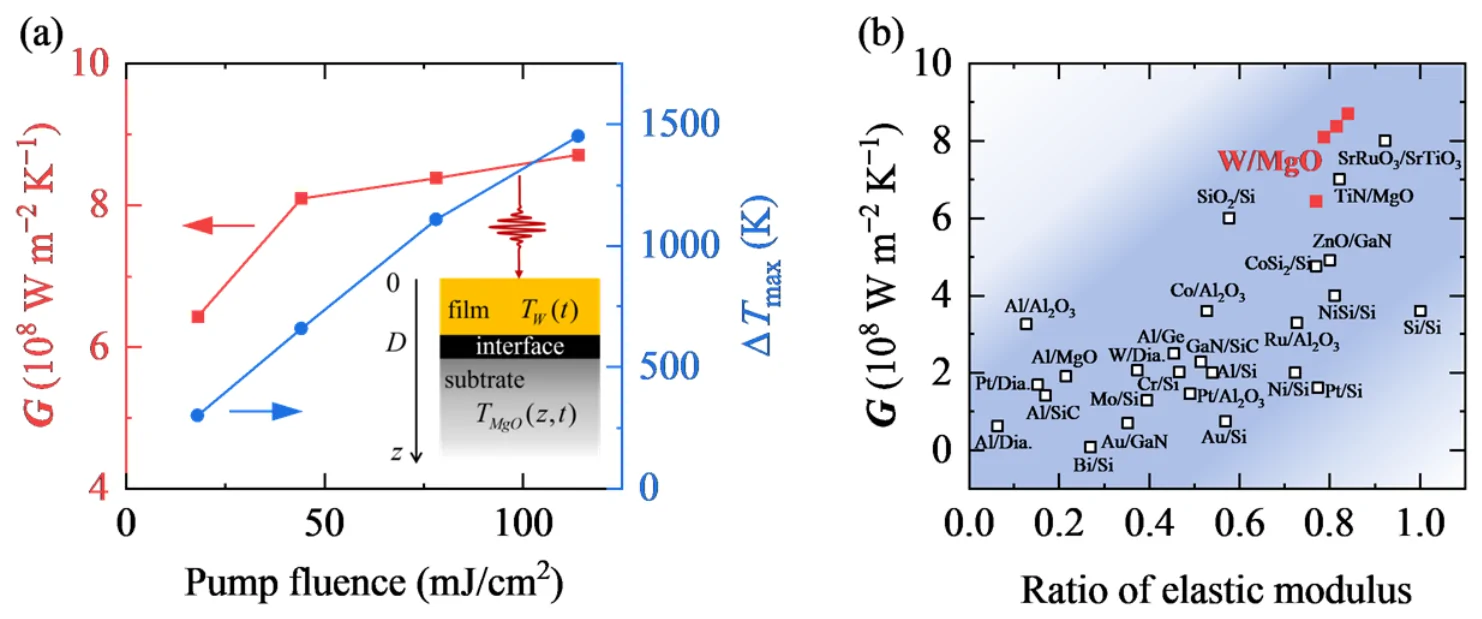
(**a**) Pump fluence dependence of the ITC of W/MgO (left axis, red line) and the maximum interface temperature difference (right axis, blue line). The inset illustrates the schematic of the one-dimensional heat transport configuration. (**b**) ITC versus ratio of the elastic moduli of the two constituents. Red solid squares represent results of W/MgO in this work. Open squares are literature results of other interfaces [16,24,32].

As shown in Figure 6a, the fitted G increases from 6.43 × 10^8^ W m^−2^ K^−1^ to 8.71 × 10^8^ W m^−2^ K^−1^ as the pump fluence rises. Meanwhile, the maximum interface temperature difference increases from 302 K to 1451 K, as revealed by the FVM. Figure 6b compares the ITC of W/MgO with typical interfaces, plotted against the ratio of elastic modulus of the two constituents. The W/MgO system resides in the upper-right region of the plot, exhibiting a relatively high ITC among the reported metal/oxide systems [16,24,32]. This result indicates efficient thermal transport across the W/MgO interface under ELTD. This may originate from the simultaneous increase in interface temperature difference and mean interface temperature.

## 4. Conclusions

In summary, we employ a table-top UXRD system to investigate thermal transport of W/MgO under ELTD. We decouple the thermal and acoustic wave contributions by fitting the temporal evolution of diffraction peak shifts. Combining with a one-dimensional transient heat transport model constructed via the FVM, we determine that the ITC of W/MgO ranges from 6.43 × 10^8^ W m^−2^ K^−1^ to 8.71 × 10^8^ W m^−2^ K^−1^ as the maximum temperature of W rises from 670 K to 2334 K, alongside a rise in the maximum interface temperature difference from 302 K to 1451 K based on the FVM. Our results show a relatively high ITC of W/MgO among the reported metal/oxide systems [16,24,32], indicating efficient thermal transport across the W/MgO interface, which may originate from the simultaneous increase in interface temperature difference and mean interface temperature. We verify that UXRD measurement is an effective strategy for probing interfacial thermal transport under ELTD. The UXRD measurement overcomes the limitations of optical thermoreflectance methods and enables a direct tracking of lattice temperature under ELTD. This work offers practical guidance for thermal management design and performance optimization under extreme conditions.

## Data Availability

The data that support the findings of this study are available from the corresponding author upon reasonable request. The raw datasets are retained to support other ongoing investigations and are not publicly available at this stage.

## Abbreviations

The following abbreviations are used in this manuscript:

ELTD	extremely large temperature difference
ITC	interfacial thermal conductance
UXRD	ultrafast X-ray diffraction
FVM	finite volume method
XRR	X-ray reflectivity
BS	beam splitter
HWP	half-wave plate
OAP	off-axis parabolic
EPC	electron–phonon coupling
TTM	two-temperature model

## Appendix A

**Table A1:** Layer structural parameters obtained from XRR fitting.

**Material**	**Thickness (nm)**	**Density (g/cm** **^3^** **)**	**Roughness (nm)**
MgO	8.99 ± 0.05	2.61 ± 0.05	0.60 ± 0.02
W	82.0 ± 0.5	20.32 ± 0.07	0.171 ± 0.004
MgO (substrate)	∞	3.58	0.88 ± 0.09
